# Refeeding syndrome in adults with celiac crisis: a case report

**DOI:** 10.1186/s13256-018-1566-6

**Published:** 2018-01-31

**Authors:** Sonia Hammami, Houda Lazreg Aref, Messouda Khalfa, Ines Kochtalli, Mohamed Hammami

**Affiliations:** 1 Department of Internal Medicine, CHU. F. Bourguiba, Monastir, Tunisia; 2 Biochemistry Laboratory, LR12ES05 Nutrition-Functional Foods and Vascular Health” Faculty of Medicine, University of Monastir, Monastir, Tunisia; 3 Department of Internal Medicine, University Hospital F. Bourguiba, Monastir, Tunisia

**Keywords:** Celiac crisis, Refeeding Syndrome, Celiac disease, feeding

## Abstract

**Background:**

Refeeding syndrome is a rare and life-threatening pathology with polyvisceral manifestations occurring in severely malnourished patients. It is rarely described in adults with celiac disease.

**Case presentation:**

We report the case of a 28-year-old Tunisian woman followed up for celiac disease, who did not adhere to the gluten-free diet. She presented to our hospital with celiac crisis manifested by severe diarrhea, and metabolic and electrolyte disturbances. The treatment of electrolyte abnormalities, hydration, and nutritional support was marked by the occurrence on the fifth day of refeeding syndrome with psychomotor agitation followed by respiratory distress and a state of cardiogenic shock.

**Conclusions:**

Refeeding syndrome is still under-recognized. It should be systematically prevented for high-risk patients. Nutritional support in patients with celiac crisis should be monitored carefully since the risk of refeeding syndrome is very high with a poor prognosis.

## Background

Refeeding syndrome (RFS) encompasses all clinical symptoms and metabolic disturbances that occur during re-nutrition in patients with chronic malnutrition [1]. If the risks of undernutrition are well known, those of re-nutrition are less common, and may lead to a syndrome of multiple organ failure [2]. In this study, we report the case of a patient with celiac crisis at the stage of severe malnutrition who developed RFS.

## Case presentation

**Fig. 1:**
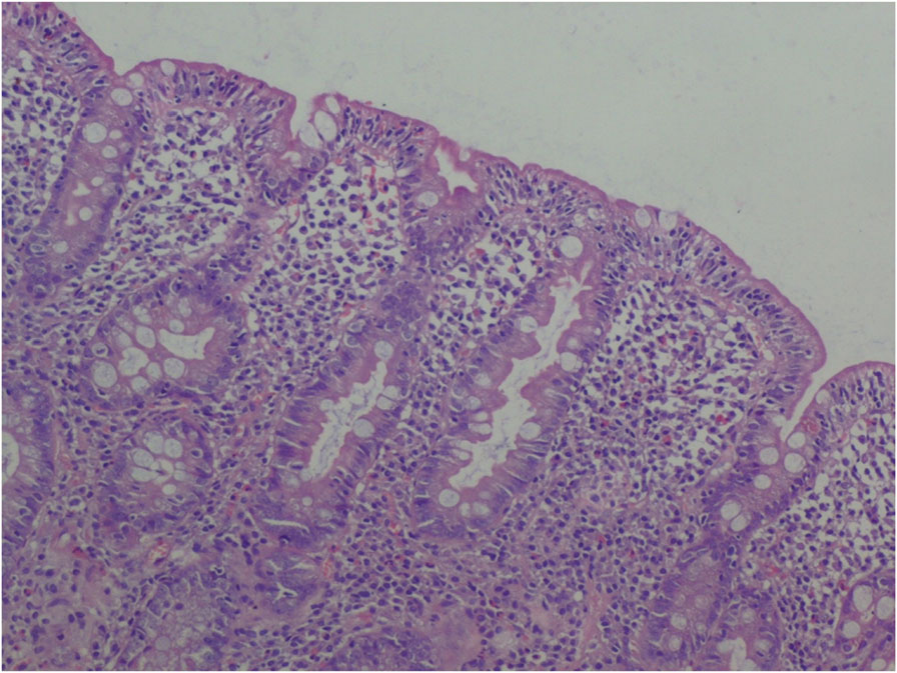
Villous atrophy with increased intraepithelial lymphocyte count (hematoxylin and eosin ×100)

A 28-year-old Tunisian woman had been followed up for celiac disease since the age of 7. The diagnosis of celiac disease was established by the positive results of serologic tests (tissue transglutaminase immunoglobulin, anti-gliadin, and anti-endomysium antibodies). The pathology report of an intestinal biopsy revealed villous atrophy with hyperplasia of the crypts and an increased intraepithelial lymphocyte count (Fig. 1). Our patient did not adhere to the gluten-free diet. She was referred to our hospital because of severe diarrhea, with severe malnutrition, and alteration of her general health status.

On admission, an examination showed a cachexia with a body mass index (BMI) of 14 kg/m^2^, signs of severe dehydration, and hypovolemic shock. In addition, abdominal ascites were present with pleuropericarditis. Laboratory tests revealed hypoalbuminemia at 14 g/L, functional renal insufficiency with creatinine clearance of 50 mL/minute and a decompensated metabolic acidosis, and hypokalemia and hepatic cytolysis at a fourfold increase. Her calcium, phosphorus, and blood glucose levels were normal. A chest X-ray revealed bilateral pleural effusion, and cardiomegaly, and a diffuse microvoltage on the electrocardiogram.

Intravenous hydration was initiated with the correction of acid-base disorders end electrolyte disturbances. Re-nutrition in the first 2 days included osmomed infusions, and oral nutrition with a satisfactory clinical response. On the third day, a parenteral feed with perikabiven was started at a rate of 450 kcal/24 hours. On the fifth day, our patient developed psychomotor agitation followed by respiratory distress, and a state of cardiogenic shock.

Laboratory tests showed hypophosphatemia of 0.3 mmol/L, hypocalcemia of 1.54 mmol/L, hypokalemia of 1.9 mmol/L, hyperglycemia at 1.6 g/L and respiratory alkalosis (pH = 7.53, PCO2 = 19 mmHg, PO2 = 80 mmHg, HCO3- = 19 mmol/L). An echocardiograph showed a noncompressive circumferential pericardial effusion and an altered left ventricular function of 20%, with no change neither in the appearance of the electrocardiogram, or in the elevation of troponins. Mechanical ventilation and the use of inotropic drugs were started. Our patient died 2 days later secondary to multiple organ failure resulting from RFS with celiac crisis.

## Discussion

RFS is an uncommon pathology with polyvisceral manifestations occurring in severely malnourished patients receiving either enteral or parenteral artificial refeeding and may cause serious complications [1, 3]. The syndrome is complex and potentially fatal, characterized by a metabolic and hormonal shift.

This syndrome is not well identified, and inappropriately treated. The true incidence is unknown due to lack of a universally accepted definition. The incidence in internal medicine departments according to Kraaijenbrink *et al.* is about 8% [4, 5]. The identification of high-risk patients is important. These include any patient chronically undernourished or recently malnourished (through inflammatory bowel disease, celiac disease, cancer, dysphagia, anorexia nervosa, depression, alcoholism, and so on), and patients with electrolyte disturbance (low phosphate, potassium, magnesium prior to feeding). High-risk patients include those with significant weight loss, and low BMI. For those patients, nutritional repletion of energy should be started slowly.

Our patient was followed for celiac disease and, through not adhering to the gluten-free diet, she developed a severe malnutrition. On admission, she presented with celiac crisis with profuse diarrhea and severe metabolic and electrolyte disturbances. Celiac crisis is an acute fulminant complication of celiac disease, rarely described in adults and generally affecting patients with no previous diagnosis of celiac disease [6]. Clinically, this diagnosis should be considered in patients with severe diarrhea, dehydration, and metabolic disturbances.

To the best of our knowledge, few cases of RFS have been reported during gluten-free re-nutrition in celiac patients. Agarwal *et al.* reported five pediatric patients [7]. Recently, Lenicek *et al.* reported another pediatric case [8]. These patients showed a severely malnourished celiac disease with a BMI of less than 14, a hypophosphatemia, a hypocalcaemia, and a hypokalemia. During re-nutrition, various precipitating factors are identified for the occurrence of RFS: dehydration, electrolyte, and mineral disorders. To the best of our knowledge, this is the first case reported in an adult with celiac crisis [9]. Diagnosis of RFS is based on hydro-electrolyte disorders, including hypophosphatemia, hypocalcaemia, hypokalemia, and hyperglycemia. In the event of a severe deficiency, death may occur by multiple organ failure. In our patient, undernutrition was severe with severe hypoalbuminemia. The onset of hypophosphatemia, hypocalcemia, and hyperglycemia were the first signs of RFS.

The clinical signs also support this, showing congestive heart failure, sodium retention, and neurologic disorders. Cardiac involvement has been well described in the literature, and its manifestations and mechanisms are diverse. However, the occurrence of an authentic cardiogenic shock remains infrequent; this is the fifth case to the best of our knowledge [10, 11].

The use of parenteral nutrition in our patient and in this context may have aggravated cardiac decompensation because of the volume overload that it represents on an amyotrophic myocardium. This case report highlights the potential dangers of RFS and emphasizes cardiac-related complications.

The pathophysiologic processes in RFS include metabolic and electrolyte disorders. Re- nutrition re-initiates metabolic pathways; in fact, hyperglycemia stimulates insulin secretion. Insulin promotes glucose uptake, inhibits lipolysis, and stimulates the movement of extracellular potassium, phosphate, and magnesium to the intracellular compartment. Hydro-electrolyte deficits observed during RFS are responsible for the associated clinical consequences: cardiac, renal, respiratory, neuromuscular, digestive, hepatic, and even hematologic. The syndrome is potentially fatal considering these possible complications. The identification of patients who are at risk of developing RFS is important to actively prevent and treat its possible complications [12]. The criteria for identification of patients at risk were established by the National Institute for Health and Clinical Excellence (Table 1) [13].

**Table 1:** Criteria for determining patients at high risk of developing RFS

Patient has one or more of the following:
• a BMI of less than 16 kg/m2
• unintentional weight loss greater than 15% within the last 3–6 months
• little or no nutritional intake for more than 10 days
• low levels of potassium, phosphate, or magnesium prior to feeding
Or patient has two or more of the following:
• a BMI of less than 18.5 kg/m2
• unintentional weight loss greater than 10% within the last 3–6 months
• little or no nutritional intake for more than 5 days
• a history of alcohol abuse or drugs including insulin, chemotherapy, antacids, or diuretics

*BMI* body mass index, *RFS* refeeding syndrome

Our patient had chronic malnutrition. She had always refused to accept her illness and had not respected the gluten-free diet. Most of the time, celiac crisis occurs in patients without a previous diagnosis of celiac disease, or in patients with previous diagnosis of celiac disease and with dietary transgressions. Multidisciplinary management could have improved the prognosis, with collaboration between nutritionist, internist, and psychologist for better adherence to the diet and acceptance of the disease. At admission, a perfusion was initiated with enteral feeding. Supplementation with vitamins, selenium, magnesium, and cofactors such as thiamine would have been desirable; but corticosteroids as a therapeutic option for celiac crisis can aggravate the risk and contributes to the electrolyte depletion [6].

During celiac crisis, the risk of RFS should be assessed. Recent studies highlight the interest of supplementing systematically with vitamins, selenium, magnesium, and thiamine on admission and even in the absence of biological abnormality. This is important in order to limit hypophosphatemia in particular [14]. Therefore, nutrition support should be started at a rate of 5 to 10 kcal/kg/day and increased by 5 kcal/kg/day the following days, not exceeding 500 kcal/day during the first 3 days. We can gradually reach 30 to 40 kcal/kg per day in 7–10 days, depending on the severity of the malnutrition. In the present case, the administration of 450 kcal/day was metabolically fully active.

Oral administration of the same number of calories would have had less impact on the occurrence of RFS. When the patient is feeding orally, the energy and protein intake should be assessed and quantitatively and qualitatively adjusted. The monitoring of clinical and biological parameters is imperative in the management of patients at high risk. The nutritional support must be modified in accordance with the clinical and electrolyte disturbances (hypophosphatemia, hypokalemia, and hypomagnesemia) [15].

## Conclusions

RFS is poorly recognized; it should be prevented and treated during celiac crisis, based on close clinical and biological monitoring, when starting a gluten-free diet. Multidisciplinary teams can help to provide education on its prevention.

## Abbreviations

BMI: Body mass index
RFS: Refeeding syndrome
